# Microstructural Evolution of Al-Cu/TiC In Situ Composites via Solid–Liquid Titanium–Carbon Reactions

**DOI:** 10.3390/ma18235374

**Published:** 2025-11-28

**Authors:** Jan Marosz, Marcin Górny, Jerzy Morgiel, Andrzej Janas

**Affiliations:** 1 Department of Alloys and Composites Engineering, Faculty of Foundry Engineering, AGH University of Krakow, Reymonta St. 23, 30-059 Krakow, Poland; marosz@agh.edu.pl (J.M.); ajanas@agh.edu.pl (A.J.); 2 Institute of Metallurgy and Materials Science (IMMS), Polish Academy of Sciences, Reymonta St. 27, 30-059 Krakow, Poland; j.morgiel@imim.pl

**Keywords:** Al-Cu alloys, A201, titanium carbide, SHSB reaction, composites, MMCs

## Abstract

A method for synthesizing an in situ composite based on the A201 aluminum–copper alloy is proposed, utilizing a Self-propagating High-temperature Synthesis (SHS) reaction via the SHSB (Self-propagating High-temperature Synthesis in Bath) process. In this study, a novel synthesis approach is presented, involving a liquid titanium–solid carbon reaction to form titanium carbide (TiC) particles within the A201 alloy, in contrast to the typical solid–solid (Ti–C) reaction. The outcome of this process is the formation of TiC particles, which are primarily located along grain boundaries and contribute to grain refinement, particularly of the (α)Al phase. A focused study of the in situ TiC-reinforced composite was conducted using XRF, optical microscopy (OM), scanning electron microscopy (SEM), transmission electron microscopy (TEM), X-ray diffraction (XRD), and Vickers microhardness measurements. The present study has a basic research character and focuses on the description of a novel synthesis method for the production of titanium carbides. This reaction proceeds as a solid–liquid type reaction between carbon and titanium. Phase and transmission analyses confirmed the formation of titanium carbides. Furthermore, based on the A201 alloy, the potential for alloy modification was demonstrated, which may inhibit the growth of primary α-aluminum phase grains and thus reduce the susceptibility to hot cracking.

## 1. Introduction

The development of composite materials is driven by the fact that currently-used materials, such as iron or aluminum-based alloys, show limitations in key functional properties including tensile strength, resistance to abrasive wear, and the stability of these properties at elevated temperatures [1,2,3]. This is a result of the thermodynamic properties of phases that form through eutectic, monotectic, or eutectoid transformations, among others. It is well known that, particularly in aluminum-based alloys, the primary structure can be modified by introducing other phases that strengthen the matrix of the alloy, such as TiB_2_ or TiC. They are added during the casting process in the form of Al-B or Al-Ti.

“master alloys”, acting as modifiers (grain refiners). Even in small quantities, these additions alter the material’s properties by minimizing unfavorable characteristics such as hot cracking or excessive development of coarse primary structures [4,5,6,7]. In the case of the investigated A201 alloy, the described process is well-established and widely used, especially by aerospace and automotive industries [4]. The main technological issue is the fact that it is prone to hot cracking, just like the other alloys from the Al-Cu group [8,9,10,11,12].

The process of transforming the classical A201 alloy into a composite material reinforced with titanium carbide particles is based on the principle of a liquid–solid reaction. In this case, it resembles a classical exchange reaction, in which the Al_3_Ti phase present in the Al-Ti master alloy reacts with graphite to form titanium carbide (1) [13,14].

Al_3_Ti + C ⟶ 3Al + TiC (1)

However, for this process to proceed without disruption, it must be carried out in a vacuum furnace and the temperature must be raised up to a level surpassing that routinely used during casting of aluminum alloys. According to the Ti-Al phase diagram, a temperature of 1370 °C ensures the decomposition of the Al_3_Ti phase into aluminum and titanium. The addition of graphite— prepared as a specially formed briquette— into the molten metal, leads to a liquid–solid reaction. This is an unconventional yet successfully applied new version of the SHSB synthesis process, representing a modification of the method, which is based on the classic SHSB reaction described by Olejnik et al. [15,16,17,18,19]. In the latter works, a comprehensive overview of the SHS reactions used in the zone reinforcement of functional materials have been provided. Fraś et al. [20,21], however, chose a synthesis based on the classic SHSB reaction, which ensures volumetric reinforcement of the materials (mainly aluminum alloys). There are a few sources in the literature dedicated to the transformation of Al-Cu alloys into cast MMC-type composites [22,23,24]. The proposed innovative method for producing composite material has the potential to aid in the modification of the A201 alloy by refining its microstructure through the suppression of primary grain growth of the α-Al phase. It ensures good interfacial quality between the reinforcing phase and the matrix in an “in situ” composite. In ex situ cast metal composites, unfortunately, low wettability is a significant issue that requires additional treatments aimed at obtaining a homogeneous microstructure. This requires the use of appropriate active coatings and maintenance of high purity of the reinforcing phases, such as SiC [22,23,24,25]. The literature offers limited information on the potential for strengthening Al-Cu alloys—particularly high-performance types such as A201—through the formation of titanium carbides (TiC) via a liquid titanium–solid carbon reaction.

Therefore, the aim of the present work is to investigate the possibility of the development of Al-Cu/TiC in situ composites via solid–liquid titanium–carbon reactions, while preserving control of microstructure and properties of this metal matrix composites (MMCs) based on high-strength 2xx series alloys.

## 2. Experimental Procedure

### 2.1. Melting Procedure

**Figure 1:**
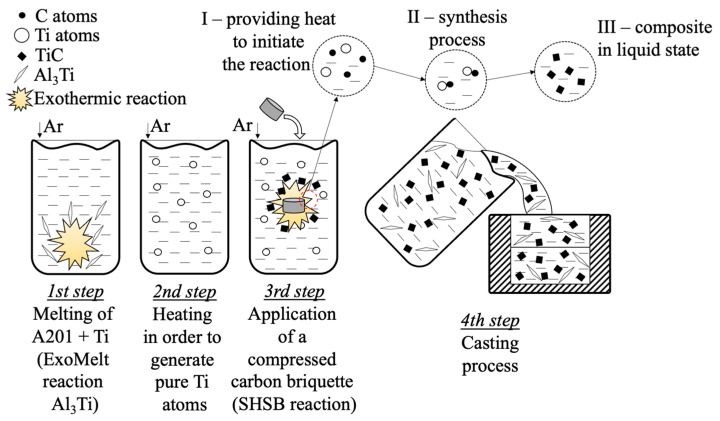
Scheme of experimental steps.

The melting of the Al201 was performed under an argon (3N) protective atmosphere with a BalzersV02 vacuum furnace, (Oerlikon Balzers, Balzers, Lichtenstein), operating at 10 kHz of an induction current frequency. The appropriate titanium content (in the granular form) for the precipitation of an Al_3_Ti phase in the A201 alloy through the Exo-Melt reaction was calculated based on equilibrium systems and the enthalpy of the formation of individual phases (Ellingham–Richardson diagram) as well as the literature data [26]. The diagram of the process is presented in Figure 1.

The experiments were divided into three stages involving preparation of

○ reference material: cast A201 alloy (reference sample);
○ A201 alloy enriched with the intermetallic Al_3_Ti phase;
○ A201/TiC composite material.

The process proceeded as follows: a proper amount of the ASTM B179-09 A201 alloy was melted under an argon atmosphere and superheated to approximately 780 °C for two minutes, then poured into a wedge-shaped chill mold.

The next stage involved the preparation of the A201 alloy enriched with titanium introduced in the form of granules. During melting, the ExoMelt (exothermic) reaction occurred, resulting in the formation of the Al_3_Ti phase. After completion of this reaction, the resulting melt was poured into a wedge-shaped chill mold and used later on as the reference alloy.

**Figure 2:**
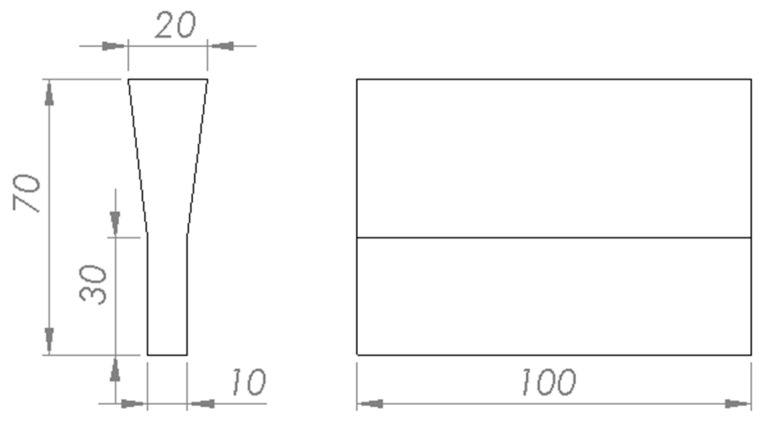
Scheme of wedge-shaped chill mold.

Next, the SHSB process was carried out to create composite material, i.e., an A201 aluminum alloy reinforced with TiC. The alloy enriched with the Al_3_Ti intermetallic phase was superheated to approximately 1400 °C to ensure the decomposition of Al_3_Ti into free Al and Ti. The furnace, equipped with a three-chamber revolver-type feeder, contained a chamber with a graphite briquette (spectral graphite). Once the desired temperature was reached, the compacted graphite briquettes were introduced. After 5 min, the temperature was lowered down to ~750 °C, and the melt was cast into a wedge-shaped chill mold (Figure 2). Prior to pouring, the mold was preheated to a temperature of ~150 °C.

**Figure 3:**
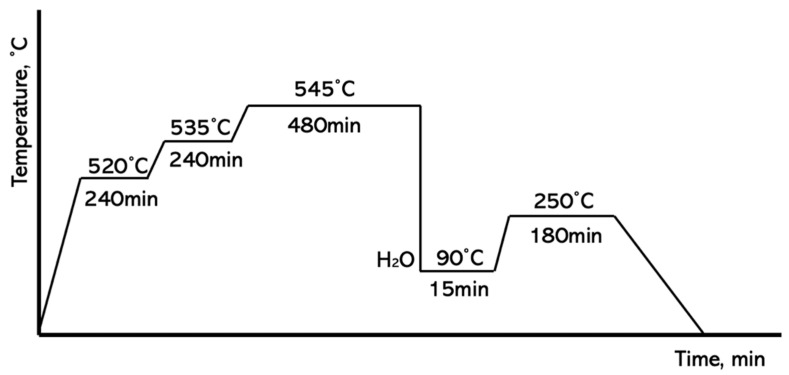
Scheme of applied heat treatment.

Al-Cu alloys are typically subjected to special heat treatments (usually T6 or T7) involving solutionizing and artificial aging. Therefore, the obtained composite material was heat-treated according to the scheme shown in Figure 3.

### 2.2. Metallography Procedure

Samples for structural analysis were taken from the central section of the lower part of the wedge-shaped castings. They were cut using a laboratory saw with simultaneous cooling. Each sample was mounted in acrylic resin. Subsequently, the samples were carefully grounded and polished. After each stage, the samples were thoroughly rinsed to remove any remaining solution. Finally, ultrasonic cleaning was employed for the thorough cleaning of the polished surface.

The metallographic observations were started using a Keyence VHX7100 light microscope (Keyence Corporation, Osaka, Japan). It helped to image the obtained metallographic sections in both the polished and etched (HBF_4_) form. The more detailed microstructure observations were performed with Tescan Mira scanning electron microscopy (Tescan Orsay Holding, Brno, Czech Republic). It allowed us to determine the micro-chemical composition in selected areas using the EDS method, along with the generation of elemental concentration maps.

Characterization of carbides were performed using a probe-corrected Themis transmission electron microscope (TEM, 200 kV) equipped with a field emission gun (FEG) and a windowless four-quadrant Super-X detector (132 eV resolution) integrated into an Energy Dispersive Spectroscopy (EDS) system (Thermo Fisher Scientific, Eindhoven, The Netherlands). Thin foils for this examination were prepared by the focused ion beam (FIB) technique with a Scios dual-beam system (Thermo Fisher Scientific, Eindhoven, The Netherlands). Elemental distribution maps representing local chemical composition were acquired with a 0.5 nm electron probe and consisted of 500 × 500 pixels based on net count data.

## 3. Results

### 3.1. Chemical Composition

The chemical composition obtained using the XRF analysis was carried out for three types of materials: the base alloy, the alloy enriched with the intermetallic phase Al_3_Ti, and the final composite material containing titanium carbides (results presented in Table 1).

The base alloy is characterized by a typical chemical composition for this material, containing ~5% copper. The titanium content in the base material, the alloy containing the Al_3_Ti phase, and the final composite material with titanium carbides remains close to 2%. Aside from titanium, no other element showed significant variation, which indicates that the only meaningful change concerned the titanium content. Magnesium, present in this alloy, is essential for subsequent precipitation strengthening during the heat treatment process.

### 3.2. Metallography

**Figure 4:**
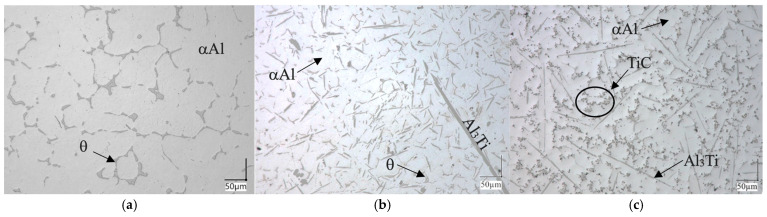
LM images of investigated materials: A201 as-cast (**a**), A201 with Al_3_Ti phase (**b**), A201 with TiC (**c**).

The aim of the experiments conducted was to present an alternative approach to the production of composite materials. According to the literature data [26], carbon is considered a detrimental element in aluminum alloys. This is because it can lead to the formation of the hydrophilic Al_4_C_3_ phase. The presence of the latter ultimately results in the material degrading over time, effectively turning it into powder. However, by ensuring the proper thermodynamic and chemical conditions, it is possible to introduce carbon in the form of briquettes, enabling the formation of titanium carbides within the alloy volume, thus producing a metal matrix composite (MMC). Figure 4a–c showed the results of three experimental variants carried out during the study.

In Figure 4a, the characteristic two-phase structure typical for alloys from the Al-Cu group is visible. The structure shows the primary phase (α-Al) and the θ eutectic (Al_2_Cu) along the grain boundaries. In the case of the second material, where the intermetallic phase was formed, needle-like precipitates of the Al_3_Ti phase were present (according to the Al–Ti phase diagram and XRD phase analysis. Occasionally, rounded precipitates of this phase were also visible, although the needle-like form predominated. Within the microstructure of the composite material, fine precipitates of titanium carbide were observed along the grain boundaries. In addition, residual precipitates of the Al_3_Ti intermetallic phase were noted, which are a result of an imbalance between carbon and titanium content. Differences between the cast sample and the composite material could also be observed, demonstrating the modifying effect on the primary (α)Al structure by inhibiting grain growth. The carbides created through the typical SHSB synthesis reaction were formed when carbon was introduced into the melt overheated to approximately 1400 °C. This reaction, described in the literature by Janas et al. [20,21,27], is a typical “solid Ti—solid C” reaction, involving the compressed non-metal powder briquettes.

The authors decided to test and demonstrate the versatility of the SHSB synthesis reaction by conducting an experiment aimed at transforming the titanium-rich phase present in the liquid aluminum alloy into titanium carbides by introducing solid carbon (2).

Ti_(l)_ + C_(s)_ ⟶ TiC (2)

where: Ti_(l)_-titanium atoms in liquid alloy; C_(s)_-solid carbon atoms, briquette.

**Figure 5:**
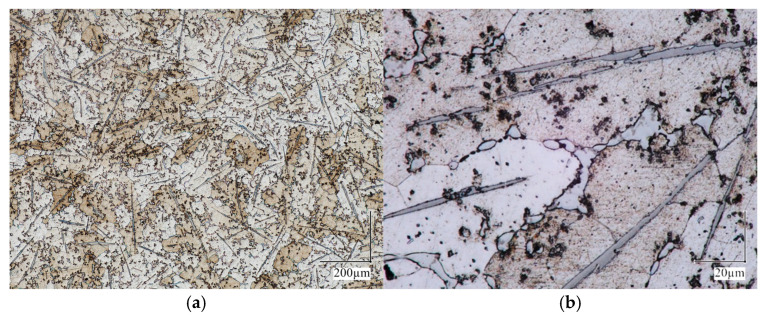
LM images of an overview of the A201/TiC composite after T6 heat treatment (**a**) and details of its microstructure (**b**).

The reaction that took place in the liquid phase involved the interaction between titanium atoms present in the molten metal and carbon atoms in the solid (briquette). As a result of this reaction, a volume-strengthened composite material based on the A201 alloy matrix was obtained (Figure 4c). The composite material sample, after heat treatment, was cut, polished, and etched using an HBF_4_ reagent to reveal the grain boundaries and the characteristic precipitation strengthening typical for these alloys (as shown in Figure 5).

The microstructure investigation showed that the titanium carbides’ morphology stayed the same throughout the heat treatment, demonstrating their high thermodynamic stability. Despite prolonged exposure of the castings to elevated temperatures followed by quenching, no dissolution of the carbides was noted. This was because the melting point of titanium carbide is around 3000 °C.

**Figure 6:**
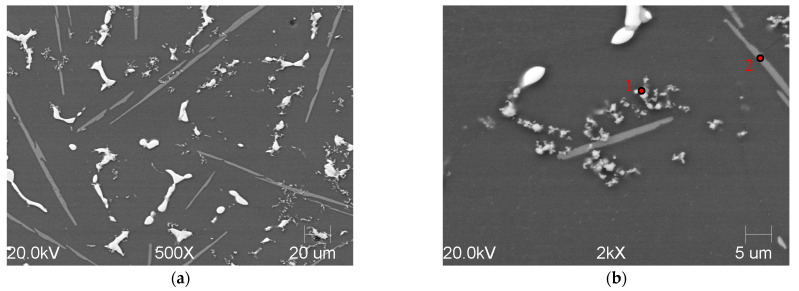
SEM/SE images of overview of A201 TiC composite (**a**) and details of its microstructure (**b**). Red points denote areas used for EDS measurements.

The results of the chemical composition analysis performed using the SEM/EDS method are presented in Figure 6 and Table 2.

It helped to confirm that the fine precipitates were typical titanium carbides (Figure 6a,b), while the needle-like phases were identified as intermetallic compounds. A slight enrichment of titanium in the matrix was also documented, but only at the level of ~0.3%. Titanium carbides were mainly located within the eutectic regions along the grain boundaries.

**Figure 7:**
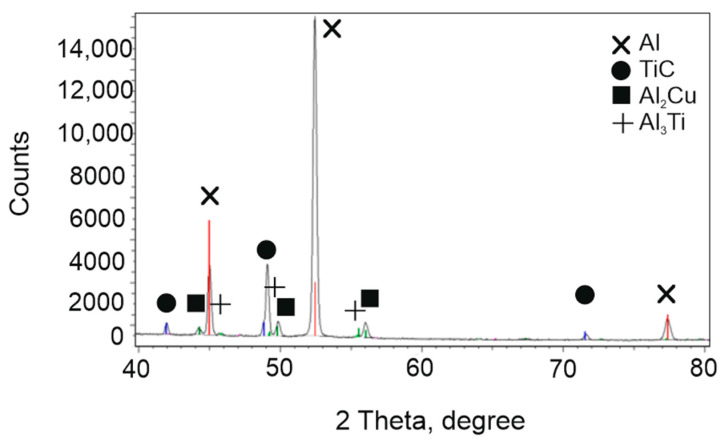
XRD spectrum acquired from A201 TiC composite.

The phase composition of the prepared composite was examined using the X-ray diffraction (XRD) method. It confirmed the presence of titanium carbide (TiC), the intermetallic phase Al_3_Ti, the aluminum matrix, and the eutectic θ phase (Al_2_Cu), which is characteristic of Al-Cu alloy systems (Figure 7).

A very interesting and noteworthy result is the absence of the Al_4_C_3_ phase, despite the addition of carbon into the molten aluminum. The presence of carbon in aluminum alloys is a significant risk due to the tendency to form this hydrophilic phase, which can lead to extensive or even complete material degradation over time. This outcome indicates that the synthesis conditions were well-optimized to promote the formation of titanium carbide (TiC) while suppressing the formation of undesirable Al_4_C_3_, thereby enhancing the long-term stability and durability of the composite material.

**Figure 8:**
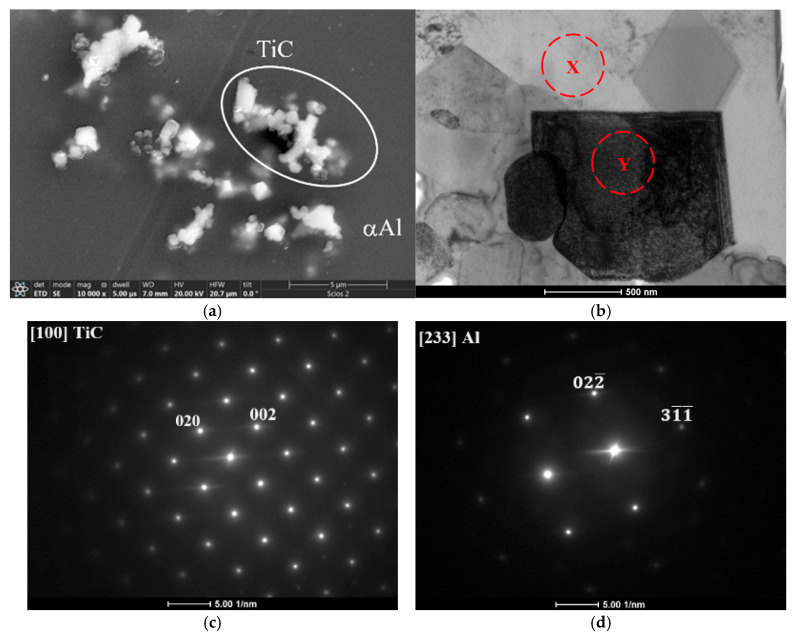
SEM/SE image presenting A201 TiC composite (**a**) and TEM/BF image of colony of TiC particles (**b**) with accompanying electron diffraction patterns (**c**) TiC and (**d**) Al, acquired from areas denoted as X and Y.

Transmission electron microscopy was employed for more precise identification of the TiC phase in the obtained composite material (Figure 8). The SEM image shows a group of particles from which the FIB lamella was prepared. This advanced method enabled a detailed description of the composite microstructure in the nanoscale, i.e., the carbides’ morphology was documented by observation in bright field (BF) mode, while their crystal structure was determined using electron diffraction. A diffraction pattern characteristic of a face-centered cubic (FCC) structure confirmed the presence of titanium carbide (TiC) in the aluminum matrix. Additionally, a diffraction pattern corresponding to the matrix itself, which confirms the identification of the aluminum phase, was presented.

**Figure 9:**
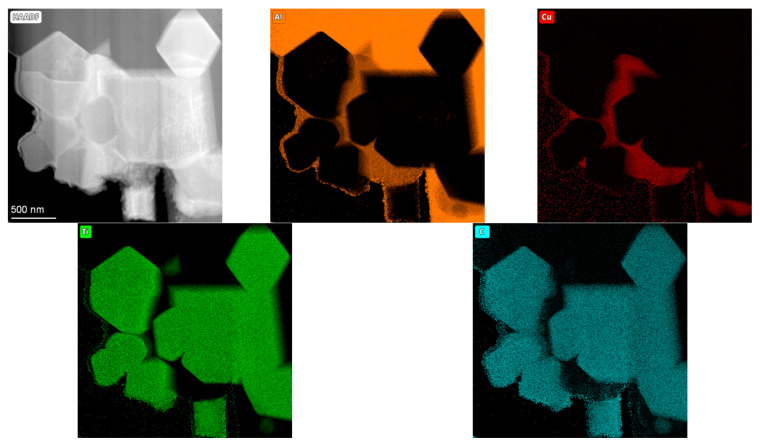
STEM/EDS image with accompanying maps presenting distribution of Al, Cu, Ti, and C.

Moreover, from the prepared lamella, an elemental composition analysis was conducted using energy-dispersive X-ray spectroscopy (STEM/EDS) and presented in the form of elemental concentration maps (Figure 9).

This allowed a precise determination of the chemical composition of both the TiC particles and the aluminum matrix, further verifying the successful synthesis and distribution of the reinforcing phases within the composite material. Additionally, these measurements showed that in this composite, the Al_2_Cu phase tends to fill in free spaces in-between the TiC carbides.

### 3.3. Microhardness

The microhardness measurements, because of the soft nature of the matrix and the relatively small size of the precipitates, were taken under a minimal load of 0.01 kgf.

**Figure 10:**
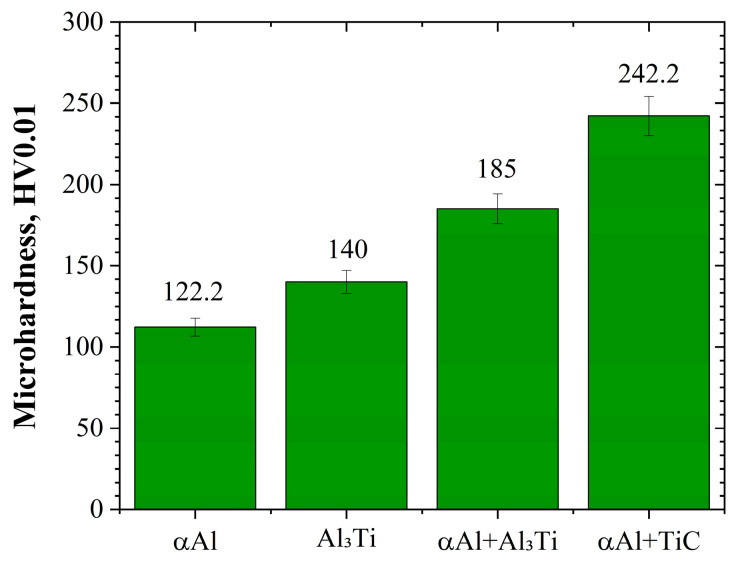
Microhardness chart for examined phases in the A201 TiC composite.

Additionally, due to the very fine, nanoscale size of the precipitates, it was not possible to measure unhindered titanium carbide hardness. Therefore, all data presented in Figure 10 should be taken as semi-quantitative ones (all except the aluminum matrix were underestimated). Simultaneously, a two-fold increase in matrix hardness was observed in areas reinforced with titanium carbide as compared to the pure (α)Al phase.

## 4. Discussion

Upon introducing titanium into the alloy, intermetallic Al_3_Ti phases were crystallized in the form of needle-like particles. Subsequently, after the addition of carbon, a synthesis reaction occurred, resulting in the formation of high-melting-point titanium carbides (whose size does not exceed 1 µm) within the aluminum matrix. Their presence was confirmed by XRD and scanning electron microscopy analysis, which further indicated that a higher titanium addition to the alloy resulted in an increased fraction of these phases, both in the casting enriched with the Al_3_Ti phase and in the composite containing TiC. TEM diffraction pattern analysis further confirmed that the fine cuboidal phases are indeed typical titanium carbides, embedded within an α(Al)aluminum matrix. Their morphology remained unchanged even after T6 heat treatment, which is attributed to its high thermodynamic stability similarly observed for the Al_3_Ti phase. The conducted microstructural analysis confirmed the reinforcing role of titanium carbide within the Al-Cu alloy matrix. Specifically, the titanium carbide particles were predominantly located along grain boundaries. Such positioning, in accordance with [26], inhibits the growth of the α(Al) phase grains, i.e., results in a grain refinement, which is particularly beneficial in terms of strengthening and the potential reduction in hot-cracking susceptibility in this group of alloys. It can be stated that titanium carbide particles form a three-dimensional interconnected structure, resulting in a nearly continuous rigid network distributed along the α-grain boundaries. Moreover, Vickers microhardness testing was performed to determine the degree of reinforcement provided by the titanium carbides within the matrix, which revealed approximately a two-fold increase in hardness in the matrix region.

**Figure 11:**
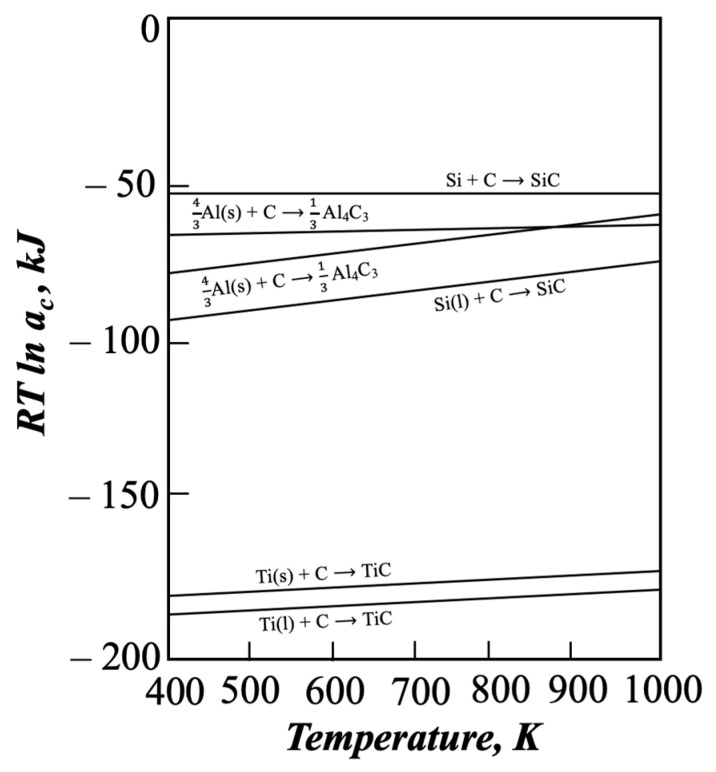
Standard free energy of carbide formation versus temperature (based on [27,28]).

The present experiment indicated that in order to avoid the formation of aluminum carbides in aluminum-based composites, it is necessary to maintain the titanium concentration in the α-Al solid solution high enough to attain a composition within the three-phase triangle consisting of Al(l) + TiC(s) + Al_3_Ti(s) [26]. Such conditions were achieved in the present study, enabling the formation of titanium carbide particles without the presence of the detrimental Al_4_C_3_ phase. The stability of titanium carbides is illustrated in Figure 11, which presents the standard free energy of carbide formation as a function of temperature.

Analysis of the standard free energy of formation shows that when a sufficient amount of titanium is added to the liquid metal, Al_4_C_3_ is replaced by the more stable TiC reinforcement. It demonstrates the universal and highly advantageous nature of the SHSB synthesis occurring between liquid titanium and solid carbon.

It is also worth mentioning the work of Cai et al. [29], who demonstrated the effect of Cd microalloying and TiC nanoparticles on the strength of Al–Cu alloys fabricated by WAAM (Wire-Arc Additive Manufacturing). In turn, Zhang et al. [30] investigated the influence of in situ synthesized TiC content on the microstructure and mechanical properties of WAAM-fabricated Al–Cu alloys, while Zhao et al. [31] analyzed the impact of TiC particles on grain boundary structure and atomic diffusion in TiC/Al–Cu composites. However, the authors of the present study adopted a different approach to titanium carbide synthesis, utilizing the relatively less known and less commonly applied SHSB reaction in aluminum alloys through a solid–liquid titanium–carbon reaction, which was also successful in improving the mechanical properties of the developed material.

## 5. Summary

The performed investigation helped to establish that it is possible to conduct a “solid C–liquid Ti” synthesis reaction in the aluminum–copper alloy A201 via the SHSB method. Simultaneously, the microstructure investigations backed by SEM/TEM/EDS measurements and XRD phase analysis confirmed the absence of the Al_4_C_3_ phase, which is considered detrimental, especially in aluminum castings, due to its hydrophilic nature. The titanium carbide reinforcing phases exhibited a structure-modifying effect by inhibiting grain growth, which may reduce the alloy’s susceptibility to hot cracking. Moreover, the close lattice parameter match between TiC (0.433 nm) and the Al matrix (0.405 nm) indicates the possibility of forming a coherent or semi-coherent interface, as the resulting 6.5% mismatch remains within an acceptable range. Finally, it should be stressed that owing to the “in situ” nature of the composite production method, high-quality bonding is ensured at the reinforcing phase–matrix interface, which further supports the effectiveness and applicability of the proposed approach.

## Figures and Tables

**Table 1:** Chemical composition for the investigated A201-based alloys obtained from XRF measurements.

Alloy	Chemical Composition, %wt.					
	Cu	Mn	Mg	Fe	Ti	Al
A201 as-cast	4.94	0.67	0.01	0.04	0.22	Bal.
A201 Al_3_Ti	4.98	0.65	0.01	0.03	2.40	Bal.
A201 TiC	4.88	0.69	0.01	0.02	2.30	Bal.

**Table 2:** Results of local chemical composition measurements performed on A201 TiC alloy using SEM/EDS.

Spot	Chemical Composition, %wt.				
	Al	Ti	C	Cu	Mn
1	37.8	34.2	26.5	1.4	0.1
2	91.2	0.3	5.5	2.7	0.3

## Data Availability

The original contributions presented in this study are included in the article. Further inquiries can be directed to the corresponding author.

## Abbreviation

The following abbreviations are used in this manuscript:

A201 base	Al-Cu (A201) alloy as cast
A201 Al3Ti	Al-Cu (A201) with Ti3Al particles
MMCs	Metal Matrix Composites
SHSB	Self-propagating High-temperature Synthesis in Bath
A201 TiC	Al-Cu (A201) reinforced with titanium carbides by SHSB method
